# Occupational hand dermatitis in car repair workers

**DOI:** 10.3934/publichealth.2019.4.577

**Published:** 2019-12-17

**Authors:** Mohammad Javad Zare Sakhvidi, Ziba Loukzadeh, Hamid Dehghan Tezerjani

**Affiliations:** 1 Department of Occupational Health, Shahid Sadoughi University of Medical Sciences, Yazd, Iran; 2 Industrial Diseases Research Center, Center of Excellence for Occupational Medicine, Shahid Sadoughi University of Medical Sciences, Yazd, Iran; 3 Yazd Rural Water and Waste Water Company, Yazd, Iran

**Keywords:** dermal exposure, dermatitis, occupational health, contact dermatitis, repair workers, mechanic

## Abstract

**Introduction:**

Exposure to used gasoline engine oils during oil change and other automobile repair services is common for many mechanics, electrical technicians, and other car service workers. We aimed to determine the prevalence of hand dermatitis in car repair workers with different specialty and actual dermal exposure hazards in the workplace.

**Methods:**

We examined the dermal problems in 153 male car repair workers and compared it to 140 office workers. Exposed and control groups were administered a Nordic Occupational Skin Questionnaire. Dermal exposure score also was calculated.

**Results:**

The prevalence of hand dermatitis in car repair workers (19.0%) was significantly higher than office worker (7.9%) [OR: 2.74, (95% CI = 1.31, 5.73)] and also higher than general population. Prevalence of atopic dermatitis was significantly higher in exposed group that had hand dermatitis compared with those who had no hand dermatitis (P < 0.001). The highest hand dermatitis as well as actual dermal exposure was observed in the mechanics and transmission technician respectively.

**Conclusion:**

Car repair workers have an elevated prevalence of hand dermatitis in comparison with office workers. The most important risk factors for hand dermatitis among car repair workers are atopic dermatitis and the next the level of skin exposure to potential skin hazards.

## 1. Introduction

Occupational contact dermatitis (OCD) is an inflammatory response caused by contact with irritant or allergen factors in the workplace. The OCD is one of the most common occupational diseases. The incidence rate of OCD is around 0.5–1.9 cases per 1000 full-time workers [1],[2]. However, in most of statistics, the mild skin problems are not being registered correctly and therefore the reported incidence of OCD is being underestimated [1]. In addition to the host related factors (such as atopy), external environmental factors (such as handling of chemicals, wet work conditions, and work practices) play an important role in the development of OCD [3].

Automotive and motorcycle technician (hereafter in the text AMTs) are at an increased risk of developing both of allergic and irritant OCD [4]. Dermal exposure is responsible of about 80% of exposures to chemicals among AMTs [5]. In addition, OCD is responsible of about 55% of reported skin disorders in this group of workers [6]. AMTs are usually exposed to both skin irritants (such as fuels, solvents, various oils and skin cleaning agents and used gasoline engine oil), and allergens (such as metals, rubber, preservatives and additives) [6]. Despite dermal exposure to hazards described for AMTs, several studies reported low level of protective behaviors and use of personal protective equipment's in AMTs. Low level of protection among AMTs could lead to an increase in dermal problems. Decrease in manual dexterity due to use o gloves or hard nature of the job are among the most reported reasons for this unsafe behavior [4],[6]. Additionally, lack of knowledge about potential harmful effects of dermal exposure to hazardous agents at the workplace is another important cause of low level of skin protection in AMTs.

There are limited studies about occupational skin diseases in AMTs. However, most of available studies are in European and North American workers. Considering the ethnic differences in susceptibility to contact dermatitis, and also different protective behavior across cultures; it is necessary to consider prevalence and pattern of the problem in other communities [7]. In this study, we aimed to a). determine the prevalence of OCD in a group of AMTs with different specialty, b). determine the frequency of using personal protective equipment, c). evaluated some risk factors associated to OCD and its relation to dermal exposure level and d). investigate AMTs actual dermal exposure hazards in the workplace and its relationship with the observed skin disorders.

## 2. Materials and methods

### 2.1. Study design

This cross-sectional study was conducted on 153 AMTs and 140 office workers in Yazd, Iran. Totally 153 male AMTs in six job categories a) car mechanic, b) transmission technician, c) electrical systems worker, d) oil service technician, e) drivability technician, f) motorcycle mechanic and g) miscellaneous job categories (including body repair and painting workers) were selected as an exposed group.

### 2.2. Sample size and sampling method

Sample size calculation was based on the results of pilot survey on 10 mechanics with considering power of 0.8 and α = 0.05 [8]. Exposed group participants had at least one year history of regular exposure to the chemicals on a defined job categories. Because the prevalence of hand dermatitis in males office workers is not significantly different from the males general population [9], 140 office workers who never had history of occupational exposure to irritants or allergens were selected randomly as a non-exposed group. Exposed and non-exposed groups were matched according to age, educational level, smoking habits, weekly working hours, and working history in years.

### 2.3. Data collection

Exposed and non-exposed groups were administered a questionnaire at their workplaces. A questionnaire prepared from Nordic Occupational Skin Questionnaire (NOSQ–2002) [10], and a validated questionnaire developed by Smitt et al. [11]. The questionnaire included demographic characteristics (education, occupational history, etc.), occupational characteristics (handling of chemicals, exacerbating factors, hand washing and use of various agents for hand cleaning, use of protective gloves), and dermal diseases history (history of atopic dermatitis, skin symptoms of hand dermatitis during the past 12 months and their connection to the workplace and severity of the skin symptoms). Hand contact dermatitis was assessed by using a Smitt et al. symptoms based on data gathered by the questionnaire [11]. OCD was defined as a positive answer to at least two of the five following items (1a–1e) in first question and a positive answer to either second or third questions. In this study, we did not make a distinction between irritant and allergic OCD, because similarity in morphologic features of both.

Dermal exposure score of each participant (in the exposed group) was calculated according to the DREAM Method [12],[13]. Data gathering was performed by a skilled occupational hygienist observer using a modified DREAM questionnaire and entered into a structured sheet. In DREAM method, relevant data such as physical state, concentration, rout of dermal exposure (emission, deposition, and transfer), and protective factor for hands and body were recorded. Protective factor for the type of clothing or gloves, frequency of replacement, duration of contact with hands, wearing gloves duration, wearing suitable gloves, put on the second glove on the first glove, how often to replace the gloves and use of protective cream was also determined. The actual dermal exposure score was calculated by taking into account the protective clothing factor for the hand and the rest of the body. Detailed procedure of DREAM exposure assessment is available elsewhere [14].

### 2.4. Data processing

Descriptive statistics were performed for all subjects according to the demographical and working characteristics. The distribution of continuous variables was examined with Kolmogorov-Smirnov test. Non-parametric tests were used in the case of deviation from a normal distribution. Qualitative data were assessed using Chi-square test and student t-test was used to compare means. Kruskal-Wallis Test was used to compare values of skin exposure between occupational groups and to compare values of skin exposure in subjects with and without hand dermatitis. To control the effects of potential confounders (i.e. second job and smoking) on the comparison of hand dermatitis prevalence between car repair workers, logistic regression was used. The significant level was considered at less than 0.05 in the study. SPSS software package version 13 (SPSS, Inc., Chicago, IL), was used for statistical analysis.

### 2.5. Ethical consideration

The study was approved by the ethics committee of Shahid Sadoughi University of Medical Sciences. All study participants were informed about the aims of the study and signed the informed consent. The data collected were kept confidential.

## 3. Results

In the present study, 153 AMTs as an exposed group and 140 non-exposed were evaluated for hand dermatitis and underlying factors. Exposed group was comprised from 33 (21.6%) car mechanics, 15 (9.8%) transmission technician, 25 (16.3%) oil service technician, 17 (11.1%) drivability technician, 23 (15.0%) motorcycle mechanic, 21 (13.7%) electrical systems worker and 18 (12.5%) others job categories (body repair and painting workers). Table 1 shows the demographic characteristics of participants. The mean age of the exposed group was 32.79 years. Exposed and non-exposed groups were comparable according to the age, working experience and marital status. Smokers in the exposed group were significantly higher than non-exposed group. However, 10.7% of non-exposed subjects had second job which was significantly higher than the exposed group which only 3.3% of them holding second job.

**Table 1.** Demographic characteristics of exposed and non-exposed group.

Parameters	Exposed group (N = 153)	Non-exposed group (N = 140)	p-value
Age (years)^1^	32.79 ± 9.46	31.98 ± 9.60	0.32
Work experience (years)^1^	13.59 ± 10.58	12.97 ± 8.36	0.09
Smoking (yes)^2^	36 (23.5)	5 (3.6)	< 0.001
Second job holding (yes)^2^	5 (3.3)	15 (10.7)	0.011
Marriage(married)^2^	105 (68.6)	101 (72.1)	0.43

^1^Mean ± SD: *t*-test; ^2^N (%): Chi-square test.

The prevalence of hand dermatitis within the past 12 months in the exposed group (19.0%) was higher than the non-exposed group (7.9%) with a relative risk of 2.74 (95% CI = 1.31, 5.73). After controlling for possible confounders, the adjusted odds of contact dermatitis in car repair workers was 2.47 (95% CI = 1.13, 5.38). There was no significant difference in prevalence of atopic dermatitis and hand washing frequency in the exposed and non-exposed participants (Table 2). Among the exposed group who had hand dermatitis within the past 12 months, 13.7% of them reported that their hand dermatitis improved during times were off work. About 30% of the exposed group required medical attention for their condition.

There was no significant correlation between hand dermatitis and educational level (P = 0.69) and marital status (P = 0.58). However, in terms of demographic and occupational factors, car repair workers with and without hand dermatitis, were not significantly different from each other (Table 3). However, prevalence of atopic dermatitis was significantly higher in AMTs that had hand dermatitis compared with those who had no hand dermatitis (P < 0.001). Table 3 gives detailed prevalence of hand dermatitis in car repair workers and controls according to various factors. The actual dermal exposure of workers who had hand dermatitis was higher, but we did not find a significant association between hand dermatitis and actual dermal exposure (P = 0.65).

**Table 2.** Comparison of hand dermatitis prevalence, history of atopic dermatitis and hand washing frequency in automotive and motorcycle technician and control group.

		Exposed group (N = 153)	Non-exposed group (N = 140)	p-value^1^
Hand dermatitis in the last 12 months		29 (19.0)	11 (7.9)	0.01
History of atopic dermatitis		32 (20.9)	30 (21.4)	0.51
Hand washing frequency Per day	0–5	49 (32.0)	35 (25.0)	0.48
	6–10	58 (37.9)	61 (43.6)	
	11–20	33 (21.6)	30 (21.4)	
	> 20	13 (8.5)	14 (10.0)	

^1^: Chi-square test

**Table 3.** Comparison of demographic and occupational factors among car repair workers with and without hand dermatitis.

	Car repair workers with hand dermatitis (N = 29)	Car repair workers without hand dermatitis (N = 124)	p-Value
Age (years)^1^	59.34 ± 11.19	59.79 ± 10.33	0.84
Work duration (years)^1^	13.61 ± 9.88	13.66 ± 10.76	0.98
Work time (hours/week)^1^	61.64 ± 10.88	57.84 ± 15.45	0.22
Smoking^2^	8 (27.6%)	28 (22.6%)	0.63
Atopic dermatitis^2^	14 (48.3%)	18 (14.5%)	< 0.001
Using protective gloves regurarly^2^	3 (10.3%)	21 (16.9%)	0.48
Hand washing frequency/day^2^:			0.56
≤ 10	22 (75.9%)	85 (68.5%)	
> 10	7 (24.1%)	39 (31.5%)	
Type of hand washing material^2^:			0.17
liquid soap with abrasives	15 (51.7%)	63 (50.8%)	
liquid detergents	10 (34.5%)	36 (29.0%)	
Organic solvents	0 (0%)	5 (4.0%)	
Skin exposure^3^	21.5 (20.0)	14.6 (14.0)	0.65

^1^Mean ± SD: *t*-test; ^2^N (%): Chi-square test; ^3^Kruskal-Wallis Test and median (IQR).

The highest actual dermal exposure was observed in the mechanics and transmission technician respectively (Table 4). There was a significant negative correlation between educational status as an ordinal variable and DREAM score (r = −0.18, p = 0.03). There was no significant correlation between age, experience, weekly working time and above mentioned dermal exposure scale (p > 0.05). Among the exposed group, the highest prevalence of hand dermatitis was observed in mechanics (37.9%) and transmission technician (20.7%) respectively. In logistic regression analysis between car repair workers, hand dermatitis was found to be significantly associated with atopic dermatitis (P = 0.007).

Protective gloves were used regularly only by 15.7% of the exposed group. 57.5% (n = 88) of the exposed group reported that they never used protective gloves during their working history. However, there was difference in glove usage pattern in different job categories. Oil service technicians and “other jobs” had the highest glove usage in comparison with other job categories. However, there was cases with plastic glove (n = 15, 9.8%), latex and leather gloves. In terms of the type of material used for hand washing, 51.0% of the exposed group used liquid detergents and 30.1% of them used liquid soap containing abrasives (Table 5). Organic solvents were used only by 3.3% of cases.

**Table 4.** Comparison of prevalence of hand dermatitis and actual dermal exposure scores (according to DREAM method) in different car repair workers groups.

Job categories	Prevalence of hand dermatitis N (%)	Dermal exposure (Mean ± SD)
Car mechanics	11 (37.9)	44.64 ± 31.81
Transmission technician	6 (20.7)	44.85 ± 24.53
Motorcycle mechanic	3 (10.3)	36.10 ± 27.39
Oil service technician	1 (3.4)	16.61 ± 16.12
Drivability technician	2 (6.9)	10.79 ± 8.69
Electrical systems worker	3 (10.3)	16.36 ± 22.35
Other jobs	3 (10.3)	
P-Value	0.026	< 0.001

**Table 5.** Frequency and type of hand washing material in car repair workers.

Job category	Cleaner				
	Solvents and Gasoline N (%)	Washing machine powder and sawdust N (%)	Liquid Soap N (%)	Washing machine powder N (%)	Don't know N (%)
Car mechanics	0(0.0)	21(63.6)	12(36.4)	0(0.0)	0(0.0)
Transmission Technician	0(0.0)	11(73.3)	3(20.0)	1(6.7)	0(0.0)
Oil service Technician	0(0.0)	11(44.0)	10(40.0)	2(8.0)	2(8.0)
Drivability Technician	1(5.9)	6(35.3)	7(41.2)	3(17.6)	0(0.0)
Motorcycle Mechanic	1(4.3)	17(73.9)	5(21.7)	0(0.0)	0(0.0)
Electrical systems Worker	0(0.0)	11(52.4)	7(33.3)	2(9.5)	1(4.8)
Other jobs	0(0.0)	14(77.8)	4(22.2)	0(0.0)	0(0.0)

## 4. Discussion

In this study, we found that automotive and motorcycle technician are at higher risk of dermatitis in comparison with office workers. In the present study an overall prevalence of hand dermatitis within the past 12 months among automotive and motorcycle technician was significantly higher than office workers (19% vs 7.9%) and also general population (2–10%) [9].. Dermal exposure and hand dermatitis prevalence was significantly higher in mechanics and gearbox workers compared to other automotive and motorcycle technician groups. However, our finding on 12-months prevalence of self-reported hand dermatitis was almost similar to car repair workers in other studies. Two surveys in Sweden reported the hand dermatitis prevalence of 15% and 24% [6],[15]. However, our study indicated lower prevalence than some previous studies that has been done in Norway (46%) and Egypt (32.2%) [16],[17]. This wide range of hand dermatitis prevalence (between 15% and 46%) in different studies could be relate to differences in the working conditions, research methodology, dermatitis definition and different criteria in identification of cases of dermatitis [16],[18].

There was no significant difference between the car repair workers with or without hand dermatitis in terms of risk factors for contact dermatitis such as use of protective gloves, hand washing frequency, type of hand washing material except atopic dermatitis. Atopy is the best known endogenous factor that causes a considerable risk of developing hand dermatitis [19]. In the present study, history of atopic dermatitis was found in 48.3% of car repair workers with hand dermatitis compared to those workers without hand dermatitis (14.5%). Atopic background plays an important role in the development of hand dermatitis among car repair workers. The higher prevalence of hand dermatitis in the workers with atopy background in our study is consistent with findings of other studies[1],[16]. Another survey on car workers found higher prevalence of contact dermatitis in workers with childhood eczema compared to workers without childhood eczema (25% vs 14%) but the difference was not statistically significant [6]. It seems that it is possible that those who have atopic dermatitis, tend to avoid some occupations.

Prevalence of hand dermatitis as well as actual dermal exposures was significantly higher in drivability and transmission technicians than other groups of car repair workers. The actual skin exposure of workers who had dermatitis, have been higher, however, this relationship was not significant; this indicates that due to car repair workers' exposure to a variety of irritants and allergens, they are at increased risk of developing hand dermatitis. Using organic solvents and the next level were hand washing and contact with oil and grease were important things that worse contact dermatitis in our car repair workers, also, a significant number of our study car workers were used liquid soap containing abrasives. In other study, 75% of car repair workers were also used soaps containing abrasives [6]. In a survey conducted by Attwa et al. frequent hand washing was one of important thing that worsen contact dermatitis in car repair workers and 85.7% of car repair workers were reported contact with tar and engine oil [16]. Excessive hand washing with detergents or using washing materials such as solvents, mineral oils or industrial detergents can damage the skin and aggravate contact dermatitis [16]. In another study the most common sources of irritants in vehicle and mobile equipment mechanics (n = 507) were automotive oils and fluids, solvents, oils, lubricants, fuels, lubricating oils and greases, respectively; and the common allergens are known workplace exposures for mechanics including gloves, automotive vehicles, machinery, soaps, and hand sanitizers, respectively [21].

In our study only 15.7% of car repair workers used protective gloves regularly in the workplace. In a study on 87 Egyptian car workers, none of workers did not wear gloves [16]. Jeffery et al. found that prior to onset of contact dermatitis, 10–19% of car mechanics and machinists were used protective gloves but after the identification of contact dermatitis in occupational contact dermatitis clinic, the vast majority of workers use gloves. In considering this issue, the authors concluded that car repair workers are able to perform their work tasks while wearing gloves and it is unlikely that manual dexterity would be considerably impaired by using gloves [4].

We observed 30% of car repair workers with contact dermatitis required medical attention because of the severity of skin symptoms. Our results on prevalence of repair workers need medical attention are comparable with other studies which reports about 15–30% of car repair workers need medical attention [9]. The relatively high prevalence of hand dermatitis among car repair workers compared to office workers emphasizes the need for preventive measures. An important tool for primary prevention of contact dermatitis is worker education about contact dermatitis cause, risk factors, recognition of early symptoms of contact dermatitis, safe work practice, skin care and proper use of protective gloves [16]. The role of educational activities in reducing the prevalence of occupational skin diseases in car repair workers reported in other studies [16]; As in another study, dermal exposure to PAHs in automotive mechanics after intervention (a hand washing intervention and education), has been decreased [22].

Other strategies in the prevention of contact dermatitis including identification of chemical and physical hazards, substitution with less hazardous substances and engineering controls to reduce exposure are also advisable.

Our study had some limitations. First, it was a cross-sectional study so may underestimate the true risk of contact dermatitis caused by occupational factors due to healthy worker effect, and cannot show the causal relationship. In present study diagnosis of hand dermatitis was assessed by using a symptom-based questionnaire. The finding of this study was not supported by clinical or laboratory diagnosis such as patch testing, prick skin test and IgE blood level. However, it is possible that the allergens in the standard series did not fully cover the exposure of the tested persons and the true prevalence of ACD be underestimated [6]; on the other hand it is a notion that different groups of auto mechanics differ in allergen profile and a more specific patch-test tray should be design for each one [16]. No other differential diagnostic like hand psoriasis and hyperkeratotic palmar dermatitis have been reviewed. The questionnaire-based diagnosis of hand dermatitis was validated in comparison with a clinical examination [11] and provided an adequate estimate of the prevalence of hand dermatitis [9]. A systematic review of 32 studies showed that the prevalence estimated by symptom-based questionnaires was higher than the prevalence estimated by expert opinion, except for hand dermatitis and respiratory disorders [20]. The strength of our study is that we have the quantitative data on the level of skin exposure to potential chemical hazards like PAH. Self-report may not reflect the actual pattern of dermatitis and could lead to bias in the results. Use of more objective measures and medical examination is proposed for future studies.

## 5. Conclusion

In this study we concluded that car repair workers have an elevated prevalence of hand dermatitis in comparison with office workers. The most important risk factors for hand dermatitis among car repair workers are atopic dermatitis and the next level the level of skin exposure to potential skin hazards.
